# Genetically Transitional Disease and the Road to Personalized Medicine

**DOI:** 10.3390/genes16040401

**Published:** 2025-03-30

**Authors:** Qingping Yao, Peter D. Gorevic, Greg Gibson

**Affiliations:** 1 Division of Rheumatology, Allergy, and Immunology, Stony Brook University Renaissance School of Medicine, Stony Brook, NY 11794, USA; 2 Center for Integrative Genomics, School of Biology, Georgia Institute of Technology, Atlanta, GA 30332, USA; greg.gibson@biology.gatech.edu

**Keywords:** autism, cardiomyopathy, autoimmune, genetics, monogenic, precision medicine, genetically transitional disease, variant, VUS

## Abstract

Genetically transitional disease (GTD) is emerging as a new concept in genomic medicine to straddle between the traditional binary classification of monogenic and polygenic disease. Genetic testing result reports in molecular laboratories have been predicated on the monogenic disease model, which focuses on pathogenic and likely pathogenic variants. While variants of uncertain significance (VUS) are reported by laboratories, there are challenges with regard to their clinical application so that these variants are often dismissed by ordering physicians. Unlike Mendelian disorders, where genetic variants are of high penetrance and highly probabilistic, the GTD concept is employed to highlight the impact of low-to-moderate effect gene variants whose influence on disease is modified by the genetic background. The GTD concept may explain health conditions associated with variants that are necessary but not sufficient for pathogenesis, lying in the mid gray zone between Mendelian and polygenic diseases. Although VUSs may not reach the level of pathogenicity based on American College of Medical Genetics and Genomics guidelines, they could be provisionally classified as GTD-associated variants to annotate and interpret the relationship between VUS and human genetic disease. The appropriate implementation of the GTD concept could impact patient care and research by focusing attention on the individual variability of responses in various diseases.

## 1. Introduction

### 1.1. Genetically Transitional Disease Definition

**Figure 1:**
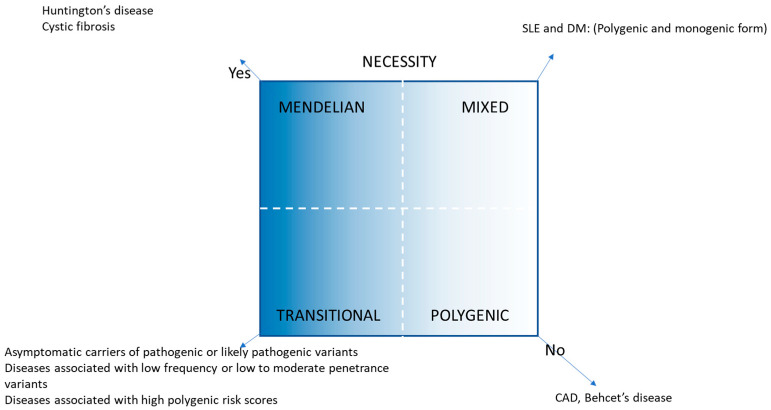
GTD and its related disease nosology [2].

There has been an explosion in the volume of genomic information over the last two decades. We have proposed genetically transitional disease (GTD) as a new concept to supplement the traditional binary classification of human genetic disease as monogenic or polygenic/genetically complex [1]. GTD refers to disease, disease status, or cases, where a gene mutation is necessary but insufficient to cause disease alone. GTD emphasizes the pervasive impact of genetic background along with the environment. Like a broad wavelength band, polygenic diseases encompass a wide range of human diseases based on genetic impact, e.g., type 2 diabetes mellitus with a weak genetic effect to Crohn disease with a larger effect [2]. GTD straddles the intermediate gray zone between monogenic and polygenic diseases and also narrows and fine-tunes the band of the latter diseases. GTD was previously exemplified with four scenarios: (1) asymptomatic carriers of a monoallelic variant in a Mendelian recessive disease (e.g., cystic fibrosis); (2) asymptomatic carriers of a pathogenic or likely pathogenic variant in Tier 1 genetic conditions (e.g., familial hypercholesterolemia); (3) diseases associated with low-to-moderate penetrance variants in variable expressivity of rare conditions (e.g., cryopyrin-associated periodic syndrome; (4) diseases associated with moderate-to-high polygenic risk scores [1,2]. Based on presumed genetic impact, GTD and its related working nosology [1] is summarized in Table 1 and Figure 1. In addition to monogenic, GTD, and polygenic disease, there is a genetically mixed form, where both polygenic and monogenic explanations for a disease may be appropriate, e.g., maturity onset diabetes of the young (MODY), in the category of diabetes [3].

### 1.2. Impact of Genetic Background and Environment on Diseases

Genetic variants are highly penetrant and deterministic in classical Mendelian disorders. For example, certain biallelic variants of the cystic fibrosis transmembrane conductance regulator (CFTR) are sufficient to cause cystic fibrosis, an autosomal recessive disease [4]. However, genetic background and environmental triggers can alter the disease expressivity (severity) and course [5]. For non-Mendelian disorders (polygenic and GTD), genetic background and environmental impact may play a more significant role in initiating and perpetuating a disease in a previously healthy individual [6]. A good example is multisystem inflammatory syndrome in children (MIS-C). It is a life-threatening complication of severe acute respiratory syndrome coronavirus 2 (SARS-CoV-2) infection, which manifests as a hyperinflammatory process with multiorgan involvement in predominantly healthy children in the weeks following mild or asymptomatic SARS-CoV-2 infection. Several gene variants or genetic defects have been identified that increase the risk of MIS-C [7,8].

### 1.3. Challenges and Opportunities in Detecting Missing Heritability

Since the completion of the Human Genome Project in 2003, hundreds of thousands of gene variants have been identified as causal agents for a wide range of rare and common diseases by Whole Exome (WES), Whole Genome (WGS), and Next-Generation (NGS) Sequencing. However, these approaches still fail to identify specific genetic abnormalities in over 50% of patients with a suspected congenital disorder [9]. Many variants of uncertain significance may be the cause of pathogenesis in some children, but due to low penetrance, there is insufficient evidence by ACMG guidelines for them to be annotated even as likely pathogenic. Challenges also exist in both the discovery and interpretation of findings from genome-wide association studies. Despite increases in statistical power with larger sample sizes, for the majority of human traits, genetic associations discovered account for a fraction of disease, limiting our ability to understand how common polymorphic risk modifies rare variant effects. Genetic variants that are outside the reach of the most statistically powered association studies are thought to contribute to the missing heritability of many human traits, including common variants (minor allele frequency [MAF] > 5%) of very weak effect, low-frequency (MAF 1–5%) and rare variants (MAF < 1%) of small to modest effect, or a combination of both [10].

GTD applies to human genetic diseases that cannot be explained by classical Mendelian segregation or fully penetrant de novo variants [2]. For example, in rheumatic disease, a subset of rheumatoid arthritis and systemic lupus erythematosus cases are associated with modest to moderate effect HLA- and non-HLA risk alleles [2]. Other examples are Crohn’s disease (40%) and Yao syndrome which are associated frequently with *NOD2* variants of low penetrance but with larger effects than standard genome-wide association study (GWAS) signals [11].

## 2. Genetic Testing Result Interpretations and Reports

### 2.1. Current Status

Since 2015, genetic testing results have been reported according to the Joint Consensus Recommendation of the American College of Medical Genetics and Genomics (ACMG) and the Association for Molecular Pathology (AMP) [12]. A variant is defined as being of “uncertain significance” (VUS) within the context of Mendelian disorders in guidelines based on functional assays, familial segregation patterns, or in silico (computational) analysis. While recognizing that genetic and genomic testing is objective and reliable, variant interpretation and classification remain somewhat subjective and even problematic, particularly with regard to VUS.

### 2.2. Limitations of Searching for High Penetrance Variants Using Monogenic Model

Genomic testing has been applied to a wide array of human genetic diseases, as well as Mendelian disorders. VUSs have been reported by molecular laboratories to account for a significant portion of the molecular results in real-world practice. The GTD concept can apply to many human genetic disorders, where genetic variants are of low-to-moderate penetrance. Unlike highly penetrant variants in Mendelian disorders, where commonly used familial and functional approaches are sufficient to characterize pathogenicity, variants strictly defined as VUS based on Mendelian disorder criteria may be elevated to clinically significant in the context of GTDs. For example, variants in the DNA mismatch repair (MMR) gene *MSH6*, identified in individuals suspected of Lynch syndrome, an autosomal dominant condition, are difficult to classify owing to the low cancer penetrance of defects in that gene. The complete in vitro MMR activity assay was calibrated against clinically classified *MSH6* variants and, employing Bayes’ rule (a mathematical rule for inverting conditional probabilities), integrated with computational predictions of pathogenicity. The two-component classification procedure, along with genetic screens, provides complementary approaches to rapidly and cost-effectively classify the large majority of human *MSH6* variants [13]. Another example is BRCA1-associated RING domain 1 (*BARD1)* in breast cancer, which is a low-penetrance gene with an unclear clinical relevance, partly because of limited functional evidence. Researchers found functional analysis of *BARD1* VUSs requires a combination of assays and, more importantly, the use of appropriate functional assays with consideration of the variant’s location in the gene [14].

To define a genomic diagnosis for a disease in a study of rare pediatric disease, candidate diagnostic variants identified were reviewed by a central clinical review panel to evaluate analytical and clinical validity. Referring clinicians then evaluated the reported variant(s), requested diagnostic laboratory confirmation where required, and communicated diagnoses to the family [15]. With the GTD concept mindset that is not limited by the monogenic disease model nor requirement for all elements of clinical presentation, a close clinical phenotype-genotype correlation for a specific disease by an experienced specialist may nevertheless reveal the clinical relevance of a VUS with a GTD, since each disease is considered by reference to presentation of one or more of specific clinical constellations of symptoms, signs, and/or laboratory findings.

Commercial laboratories generally report pathogenic and likely pathogenic variants only for Mendelian disorders; as a result, so-called “benign variants” and “VUS” (the majority are reclassified as benign) are not reported unless they are requested; however, some of them are in fact contributory to diseases. Often, ordering physicians or geneticists currently read or interpret genetic reports such that clinically meaningful variants are overlooked or dismissed [2]. This has posed challenges for gene variant filtering, calling, and interpretation. The strictly monogenic disease model used to search for causal variants of high penetrance, even with most advanced molecular technologies, currently produces diagnostic yield of most genetic testing in the range of just 15% to 39%, depending on disease category [16,17]. In other words, significant heritable factors may go unreported, and further efforts to locate and validate pathogenic variants might be limited by the adoption of current analytical approaches for Mendelian disorders. We thus advocate for inclusion of VUS where there is evidence that the variant is clearly pathogenic but with variable penetrance and expressivity. Given the potential for over-diagnosis, such reporting should include the quality of evidence in this regard.

## 3. Potential Utility of the Genetically Transitional Disease (GTD) Concept

### 3.1. The GTD Concept and VUS

The current clinically focused approach of variant classification is often underpowered to classify certain variants for some diseases. Consequently, personalized healthcare for carriers and affected relatives cannot be implemented. For this reason, it is of the utmost importance to classify these VUS [18]. To supplement the variant reports under the ACMG/AMP guidelines, GTD might be employed to highlight missing potentially causal variants so as to increase diagnostic yield. VUSs defined by traditional Mendelian approaches might be provisionally reclassified as GTD-associated variants using our approach. To avoid dismissing these variants, this approach might keep them alive for further study. These variants could be proven to be pathogenic, likely pathogenic, or benign (perhaps many cases) in the end. However, even some “benign variants” as defined based on the ACMG guidelines are contributory to diseases [19]. These conditions associated with low penetrance belong to GTDs. Experienced physicians in relevant subspecialties could perform a close correlation of phenotypes and genotypes based on specific clinical constellations particular for individual diseases. Some VUSs would be considered clinically meaningful for a particular disease, with an explicit annotation that the variant is insufficient and hence not fully penetrant, and further studies using combined functional assays and computational prediction algorithms [13,14]. For example, in cardiology, many genetic variants have been identified in dilated, restrictive, and hypertrophic cardiomyopathies, of which approximately 50% are classified as VUSs [20]. This is particularly relevant as new approaches to detecting digenic inheritance involving combinations of pathogenic and VUS variants emerge [21]. In our view, these cases may be reclassified as having GTDs. Researchers have begun to use innovative approaches such as deep mutational scanning to capture multifactorial and diverse protein function, as variants may impact pathogenicity through multiple mechanisms [22].

### 3.2. Underdiagnosis and Overdiagnosis

For the appropriate management of a disease, proper diagnosis is essential. Both underdiagnosis and overdiagnosis are disadvantageous, risky, and even consequential. A lengthy discussion of their pros and cons is beyond our main theme in this article. While there is an elevated risk of false positives, or overdiagnosis, it could be reasonably contained with more advanced and focused research. With appropriate annotation of GTD evidence, the rapid rise in WES/WGS screening that constantly updates evidence, and the availability of higher quality genomic datasets of better control populations for comparison of minor allele frequencies, this rate can be controlled. The trade-off in the inclusion of likely causal variants will also have long-term benefits. For example, based on known disease-associated gene alleles, experienced specialists will be able to correlate characteristic clinical features for a particular known disease (GTDs) with VUS alleles identified in the same gene to unveil their clinical relevance. One such example is autosomal dominant polycystic kidney disease (ADPKD), which is mainly caused by PKD1 or PKD2 gene variants. In a study on genomic diagnostics of ADPKD [23], patients were referred for diagnostic WGS, and only variants classified as ‘pathogenic’, ‘likely pathogenic’ or ‘VUS’ were reported to clinicians. Of 101 patients, 40% (40/101) carried pathogenic variants, 29% likely pathogenic, and 32% VUS, of whom 59% (19/32) of the uncertain variants favored pathogenicity. Patients with atypical phenotypes were more likely to have a VUS, and this scenario is also a good example of GTD. In addition, investigators may study a group of patients who present with a similar characteristic clinical phenotype and less specific symptoms that might not fit a Mendelian disorder. If several of these patients happen to carry the same VUS, the VUS would be likely to be of clinical relevance based on the GTD concept.

## 4. Variants of Uncertain Significance (VUS) and Interplay with Genetic Background and Environment

### 4.1. Diagnostic Consideration of Target/Candidate and Modifier Genes in Diseases

A widely recognized model of disease pathogenesis involves gene–gene and gene–environment interaction [24]. This model may apply to many human diseases. The term candidate gene refers to a gene that is thought to be related to a particular trait or disease, and can be identified by either position or function [25]. There are two types of candidate genes: classic single gene targets and those embedded in multigenic traits [26]. Examples of the former case are monogenic Mendelian disorders such as Cystic fibrosis and Huntington’s disease, where candidate gene variants play a deterministic role or are highly probabilistic in disease, although genetic background or modifier genes may also play a role [27,28,29]. Examples of the latter case are some polygenic and genetically transitional diseases, where the candidate and modifier genes also interact with the environment, which plays an equally important role [6]. In mixed NOD-like receptor-associated disease, for example, combined gene variants in NOD2 and other autoinflammatory disease genes have been identified in individual patients, where NOD2 gene variants were considered a candidate target, and other genes may be modifiers [19].

### 4.2. GTD and Digenic or Oligogenic Variants

The GTD concept can also be used in individual patients who carry two (digenic) or more (oligogenic) low penetrance variants in different genes, as more and more such variants are identified in disease cases [19]. Unlike individual variants, the frequency of combined variants identified in individual patients is not readily available in the current genomic databases such as the Genome Aggregation Database. However, a recent study [21] identified ten pairs of genes that harbor rare variants in at least half a dozen cases of congenital heart defects, but these combinations were not seen in gnomAD controls, while the highlighted genes are plausibly linked to heart development. Other genome datasets, such as the All of Us Research Program, may also be annotated to compute the combined variant frequencies and to compare these with a patient population to validate the potential impact of such combined variants in diseases. To study monogenic disease, traditional methodologies include familial segregation, case-control, population, and functional studies [30]. For GTDs without a clear inheritance pattern in most cases, familial segregation studies may have limited value.

### 4.3. GTD and Its Dynamic Nature

The GTD concept also acknowledges the evolving and dynamic nature of certain gene variants in their pathogenic role in disease, perhaps depending on genetic background and environmental triggers. In other words, variants like VUSs in silent or “healthy” status may display obvious effects in the presence of specific genetic backgrounds and/or environments. One possibility would be that GTD-associated VUSs of a gene that has been known to cause a Mendelian disorder could be proven to be pathogenic or likely pathogenic after close phenotype–genotype correlation, special functional assays, bioinformatics analysis, or other novel studies. Then further study using familial segregation approaches, including family history, pedigree analysis, and artificial intelligence analysis of genetic data of trios (parents and a child) or duos (siblings or parent-child) in family members [31] could reveal a Mendelian inheritance pattern, i.e., autosomal or recessive, or confirm that there is a significant association with disease despite incomplete penetrance. Another possibility would be that GTD-associated VUSs could turn out to be truly benign or VUS in one population context, but consequential in another [32]. We believe that the term, GTD-associated VUS, is advantageous over VUS, as it emphasizes that the gene has been associated with the disease and that attribution of causality to specific variants does not require that all carriers are affected. Hence, the term in essence keeps the variant alive and open until proven otherwise in the long run. Stating this another way, GTD emphasizes that uncertainty of significance in many cases reflects that genetic effects may be causal in an epidemiological sense (“incidence of disease is greater in people with the variant”) without being determinative for a specific case.

### 4.4. Gene x Environment Interaction

Molecular testing to search for a single pathogenic variant of high penetrance is important for diagnosis, but may not be complete. There are many potential sources of incomplete penetrance of genes, which are also important. One is gene x environment (GxE) interaction, which can be very important in the pathogenesis of disease. These types of interaction are defined as a different effect of an environmental exposure on disease risk in persons with different genotypes, or, alternatively, a different effect of a genotype on disease risk in persons with different environmental exposures [33]. Neuropsychiatric disorders have often been used for studies of G×E interactions [34]. To illustrate the GxE interaction, we take autism spectrum disorders (ASD) as an example [35]. ASD has been traditionally considered a complex neurodevelopmental disorder with a strong genetic component [36]. Rare monogenic (< 20%), modest to moderate, VUSs, and mitochondrial DNA variants have been identified in studies of ASD [36]. Variants of modest to moderate effects increase the risks of ASD [37,38], and their relative importance may be sex-biased, showing that sex is an important “exposure” to be considered in evaluating pathogenicity [39]. Many VUSs have been identified in ASD but could not be assigned pathogenicity using the monogenic disease model. Some are confirmed to increase risk to the disease, and some are suspicious [40].

Unlike Mendelian disorders, variants identified in ASD mostly act as susceptibility risk alleles for the disease with incomplete penetrance due mostly to randomness [36]. Cases of ASD can carry mitochondrial DNA deletions that coexist with other ASD-associated genetic risk factors, suggesting intergenomic communication or background gene-gene interactions [35]. In addition, environmental factors, such as prenatal and pregnancy-related infections, chemicals, and maternal comorbidities, can trigger ASD [41]. Taken together, ASD may be influenced by intergenomic and GxE interactions [42].

In our previous study of autoinflammatory diseases in adults, individual patients were identified to carry low penetrance genetic variants of two or more autoinflammatory disease genes, such as *NOD2*, *MEFV*, *CAPS3*, *NLRP12*, and *TNFRSF1A*. Based on these data, we proposed that variants of a few or several related genes could constitute a genetic background of slightly different degrees, analogous to Arabic numerals (limited numbers) used for writing codes [19]. Similarly, this hypothesis could also apply to ASD, where a number of risk alleles within hundreds of genes (mostly susceptibility alleles) are identified in patients with ASD [43], but individual patients may carry a limited number of such variants. In summary, identification of a candidate gene in disease is important, and its clinical significance should be properly interpreted and understood together with background or modifier genes within the context of the disease paradigm [24].

## 5. Discussion

Molecular technologies such as large gene panels or exome sequencing have revolutionized clinical medicine by uncovering causal variants for diseases. The identification of underlying genomic change can provide new clinical insight and support individualized therapies^,^ as well as testing and counseling [44]. The binary classification of human genetic disorders as monogenic or polygenic has been traditionally useful, but modern molecular study advances have demanded a refinement or recalibration of disease nosology [1]. The GTD concept and its working disease classification have important significance in personalized or precision medicine. As we mentioned previously, GTD could be employed to provisionally reclassify so-called VUSs or some ’benign variants’ as GTD-associated variants to pick up missing inheritability.

Future research using the GTD concept would include close phenotype–genotype correlations and creative approaches distinct from the monogenic disease model, including a bioinformatics-driven analysis of the clinical implications of such variants. Common variants of small effect identified by genome-wide association studies also contribute to diseases and may act together with rare variants [45]. Statistical genetic strategies may be employed to investigate gene x gene and gene x environment interactions that contribute to the variability of GTD rare variants, and eventually delve into the complex biological mechanisms [46]. Novel strategies to mine the clinical significance of GTD-related variants could be incorporated in the ACMG guidelines to raise diagnostic yields and benefit patient care and research while reducing health care inequality. Health disparities generally refer to systematic differences in health effects resulting from social disadvantage, but the term is often used in genomics to denote differing health outcomes associated with population genetic variation [47]. We believe that only highlighting the roles of highly penetrant variants in disease while ignoring the influence of mild to moderate variants could create health care inequality. The GTD notion will also enhance health equity as rare moderate penetrance variants will often only be observed in specific ancestry groups.

Based on the pathogenic role of gene–gene interaction in disease, the GTD concept reiterates the interaction of candidate genes along the genetic continuum, including those of low to moderate penetrance or risk alleles, with genetic background or modifier genes. Since an individual disease may harbor digenic or oligogenic variants that contribute to the disease [19,48], genomic testing of two or more variants might be utilized in a disease for diagnostic purposes rather than just targeting a single pathogenic or likely pathogenic gene in real-world clinical practice. Systemic autoinflammatory diseases (SAIDs) primarily originate from abnormal innate immune responses from mutated genes. Familial Mediterranean fever (FMF), an autosomal recessive disease, is caused by *MEFV* encoding pyrin [49]. Mutations in nucleotide-binding oligomerization domain (NOD)-like receptors contribute to diseases [50]. Cryopyrin-associated periodic syndrome (CAPS), an autosomal dominant disease, is caused by *NLRP3*, and Blau syndrome, an autosomal dominant disease, is caused by *NOD2* mutations [51]. These are typically classified as monogenic diseases due to the presence of highly penetrant mutations. However, low to moderate penetrance variants of these genes can also contribute to different diseases. In NOD2-associated diseases, for instance, the three main variants, *NOD2* R702W, G908R, and L1007fs are associated with up to 40% of cases of Crohn disease [52], and these variants are also shared by Yao syndrome, formerly designated as NOD2-associated autoinflammatory diseases [11]. These findings support the view that the same gene can give rise to a spectrum of disorders [24]. Another example of the same genotype resulting in different disease is deficiency of Suppressor of Cytokine Signaling 1 (SOCS1). Variants of differing penetrance in SOCS1 are associated with clinical constellations consistent with diseases ranging from atopic disorders, systemic autoimmune diseases such as systemic lupus erythematosus, Sjögren syndrome, and rheumatoid arthritis, to a monogenic phenotype [53]. These diseases should not be understood by a monogenic disease model only; rather, the GTD concept promotes superior and more reasonable comprehension.

Digenic variants or variants from two different genes may be understood in two kinds of scenarios. In typical digenic diseases, variants of high penetrance are inherited from two different genes and together contribute to a disease. For example, digenic inheritance in Proteasome-Associated Autoinflammatory Syndrome (PRAAS) occurs when a combination of mutations in two genes, *PSMB8* and either *PSMA3* or *PSMB4*, leads to the disease phenotype, rather than a single gene mutation [54]. Low penetrance variants from two or more different genes may contribute to diseases as well [55]. For example, combined variants in *NOD2* and other SAID genes in an individual can cause disease, mixed NLR-associated AID [19]. Therefore, genomic testing of low penetrance variants from two or more different but related genes in individual patients may be necessary and comply with the GTD concept rather than the monogenic disease model.

In our view, the GTD concept may also be used to guide therapeutic choices for diseases. For example, for breast cancer, international guidelines now classify genes as high (*BRCA1/2*), moderate (*BARD1*), and low penetrance (*BRIP1*) and favor mastectomy for high penetrance but surveillance with magnetic resonance imaging and mammograms for others [56]. It is, though, inappropriate to take a one-size-fits-all approach to such decisions as variants within genes have different expressivity and the variant should more appropriately be regarded as just one indicator of therapeutic options.

Traditional genetic counselling is used for Mendelian disorders, where there is a certain inheritance pattern and a gene variant is deterministic. Genomic counselling may be used in a wide range of disorders, including GTD and polygenic disorders, in addition to Mendelian diseases. Genetic counsellors are now required to have knowledge in multiple domains of genomics, such as genome sequencing technologies, gene variant result reports, interpretation, frequency, penetrance, and clinical impact on disease [57,58,59]. Genetic counsellors must also consider the potential interactions of candidate genes, genetic background, and environment, particularly for diseases without a Mendelian inheritance pattern. We propose that the GTD concept may be used as a new avenue for genomic counseling to supplement traditional monogenic or polygenic models in personalized medicine.

Terminology is important as it helps understand and communicate specific topics efficiently. Terminology and classification are complementary [2]. We anticipate that the GTD concept and its related nosology could have a far-reaching impact on patient care and research in the era of precision or personalized medicine.

## Figures and Tables

**Table 1:** Genetically transitional disease and working nosology of human genetic disease.

	Monogenic Disease	Genetically Transitional Disease	Genetically Complex Disease
Single gene mutation necessity	Yes	Yes	No
Single gene mutation sufficiency	Yes	Insufficient	No
Single gene mutation penetrance	High	Low to moderate	Minimal
Single gene effect size	Large	Medium	Small
Genetic background impact	Yes, small	Yes, several related genes?	Yes, multiple minor genes
Environmental triggers required	No or small	Yes, small to moderate?	Yes, large?
Examples	Huntington’s disease Cystic fibrosis	Monoallelic carriers for recessive disease; low penetrance variants for dominant disease (Cryopyrin-associated periodic syndrome with NLRP3 Q705K), Yao syndrome	Diabetes Mellitus Coronary artery disease
Inheritance pattern	Mendelian (autosomal dominant, recessive)	Atypical for Mendelian disorders or no fixed pattern	No pattern
Gene editing utility	Promising	Depending on gene penetrance	No
Genetic counseling	Traditional counseling for Mendelian disorders	Supplementary, genomic knowledge, dynamic	Multifactorial

## Data Availability

Not applicable.
